# Supplementary material for: Conditional chemoconnectomics (cCCTomics) as a strategy for efficient and conditional targeting of chemical transmission
Source: eLife. 2024 Apr 30;12:RP91927. doi: 10.7554/eLife.91927 (PMC11060718; doi:10.7554/eLife.91927)
Supplement: Figure 3—source data 3. [file elife-91927-fig3-data3.zip › Figure 3 source data3 part 2.zip!/Figure 3-Source data 3-part2/1053-1-CRISPResso_Report_mpeqg9da.zip!/CRISPResso_on_1053_2/1c.Alignment_barplot.pdf]

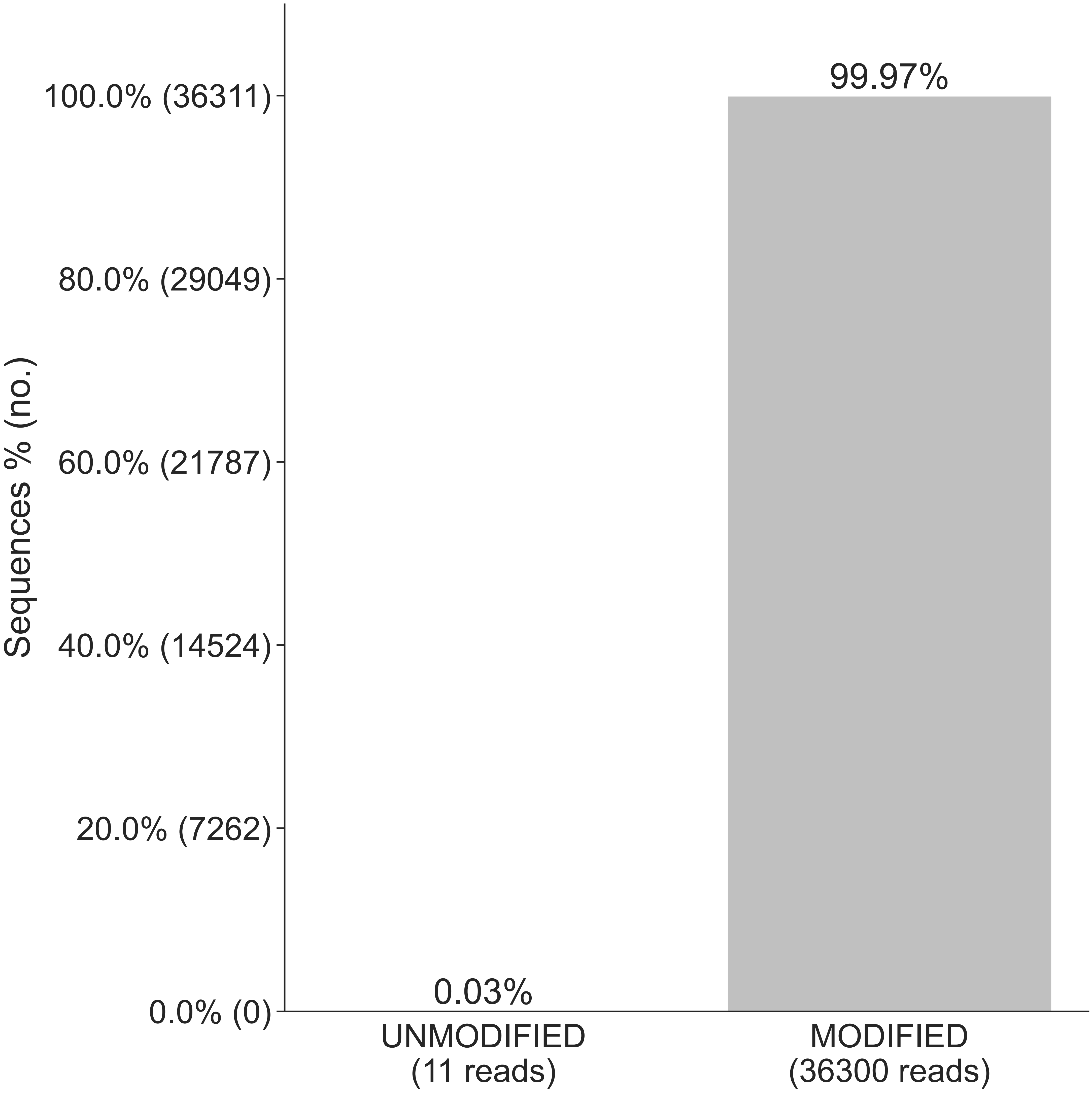

Sequences % (no.)
100.0% (36311)
80.0% (29049)
60.0% (21787)
40.0% (14524)
20.0% (7262)
0.0% (0)
99.97%
0.03%
UNMODIFIED
(11 reads)
MODIFIED
(36300 reads)
